# Poor written pragmatic skills are associated with internalising symptoms in childhood: evidence from a UK birth cohort study

**DOI:** 10.3389/frcha.2023.1075836

**Published:** 2023-06-14

**Authors:** Kalim Ahmed, Eirini Flouri, Gabriella Vigliocco

**Affiliations:** ^1^ Department of Sociology, Social Policy and Criminology, University of Birmingham, Birmingham, United Kingdom; ^2^ Department of Psychology and Human Development, UCL Institute of Education, University College London, London, United Kingdom; ^3^ Experimental Psychology, Division of Psychology and Language Sciences, University College London, London, United Kingdom

**Keywords:** language development, pragmatic language, relevance, deixis, narrative organisation, internalising symptoms

## Abstract

**Introduction:**

This study examined the relation between pragmatic language and internalising (depressive and anxiety) symptoms in 11-year-olds, using data from the 1958 British birth cohort study.

**Methods:**

The cohort children were asked at age 11 to write an essay on their life as they imagined it would be at age 25. We analysed 200 of these essays for relevance, organisation and context-dependent references.

**Results:**

We found associations between these aspects of pragmatic language and children's internalising symptom scores across parent and teacher ratings, even after adjustment for cognitive ability, socioeconomic position and structural language. Most notably, children writing more coherent essays had fewer teacher-rated internalising symptoms, after adjustment for confounders. Additionally, children who provided more relevant and varied information about their imagined future home-lives had fewer parent-rated internalising symptoms, after adjustment for confounders.

**Discussion:**

The unique associations between pragmatic language skills and internalising symptoms observed are notable but preliminary, highlighting both the need for further research and potential applications for risk-assessment tools.

## 1. Introduction

The relationship between children's language and mental health, especially emotional well-being, has attracted much research. Both language and emotional well-being are multifaceted and dynamic. For instance, language comprises receptive and expressive phonological, semantic, syntactic, and pragmatic components, and emotional well-being assumes a good level of cognitive, social and interoceptive functioning. Each changes with time, through environmental exposure, social interaction, and building on prior knowledge and abilities. Crucially, the two may be causally related, with research suggesting direct causal paths, both unidirectional and reciprocal, but also links due to shared causes. In this study we focused upon the previously unexamined relationship between written pragmatic language, the impairment of which has been seen in many neuropsychiatric disorders, and internalising (depressive and anxiety) symptoms in the general child population.

Pragmatics is the use of language in context (1). Pragmatic language abilities are thus deeply implicated in human communication and are central to social understanding. Approximately two-thirds of children with internalising symptoms or externalising difficulties (i.e., problematic behaviour related to poor impulse-control) also have pragmatic language impairments. In the psychological research to date the latter are typically measured with a composite evaluation of the child's inappropriate initiation, incoherence, stereotyped language, and poor use of context and rapport in communication (2, 3), such as the Children's Communication Checklist [CCC; (4)]. Although, arguably, difficulties with structural rather than pragmatic language (overlapping to a large extent) have attracted more interest in child psychology and psychiatry, poor pragmatic language seems to play a distinct role. For example, using a large sample of 4-year-olds, Ketelaars et al. (5), found that, once pragmatic language difficulties were accounted for, structural language deficits did not forecast internalising and externalising difficulties.

Causal paths linking pragmatic language deficits to internalising symptoms in children generally implicate poor social understanding and in turn poor social skills. Researchers who expect poor pragmatic language to be the cause rather than the effect of such symptoms argue that poor pragmatic language causes difficulties with understanding others and with self-understanding, thus leading to social isolation and loneliness. For example, children's poor social understanding can make others' intentions opaque or increase the likelihood of misattributed intentions, thus limiting their opportunities to interact and socialise with their peer groups which in turn leads to impoverished interpersonal contexts and weak social networks. Poor capacity for self-understanding can also directly lead to internalising symptoms by undermining effective emotional processing. It can also lead children to make poor choices for their needs, in turn causing or exacerbating internalising symptoms. The role of poor social understanding and as a result social isolation is also implicated in models expecting causality to run in the opposite direction, i.e., from internalising symptoms to poor pragmatic language. In this case internalising symptoms are thought to cause isolation and impoverished social contexts (and therefore poor social language skills), as well as directly skewing one's capacity for self and others' understanding *via* overly negative thinking.

It appears therefore that poor pragmatic language and internalising symptoms may be reciprocally related. However, research that has directly tested the strength of such bidirectionality in children suggests that the path from poor pragmatic language to anxiety and depressive symptoms is probably stronger. For example, Bornstein et al. [6] observed that multiple measures of language difficulties (covering semantics, syntax, production and comprehension) assessed by teacher, parent and researcher at age 4.5 years were associated with internalising (but not externalising) problems at ages 7 and 10 years, even after adjustment for non-verbal ability and various socioeconomic factors. But, internalising symptoms did not predict later language ability at any stage. Thus, poor language skills, in that study and elsewhere (7), were considered determinants rather than outcomes of internalising symptoms.

Recently however there has been a call for research to re-examine the relationship between pragmatic language and mental health in children using more refined assessments of pragmatic skills than the broad composite measures it has generally employed. This is because, as will be discussed below, pragmatics encompasses several skills at many levels, which may vary in how they are linked with emotional functioning (8–10). Our study was designed to respond to this call.

### 1.1. Which pragmatic language skills?

Pragmatic language is context-dependent and requires cooperation (11, 12). It follows then that a cooperative interlocutor is expected to produce communication that is relevant, informative and intelligible. Several factors influence one's ability to do so, from an assessment of the context and its norms to linguistic and cognitive skills. An irrelevant response to a question, for example, is very notable when it suggests a poor assessment of context. Relevance therefore may approximate contextual understanding. Arguably, a deficit in this area may bring about internalising problems by reducing the number of high-quality linguistic and social interactions, i.e., the type of interactions that can produce emotional benefits.

At a lower language level, often within-sentence, reference is another important aspect as it directs and (ideally) coordinates the foci of our attention in communication. Before words are acquired, this may be achieved through pointing (deixis). Language allows greater breadth and precision of reference, requiring abstract representations of the referent and of how accessible that referent is to one's interlocutor. Deictic references therefore require adequate monitoring and evaluation of contextual information (13). For instance, the writer/speaker of the pronoun “she” assumes that the audience is aware of the female in question. Such an ability to track and resolve references thus also reflects general cognitive development, executive functioning in particular, which is often impaired in depression. In fact, some atypical patterns of deictic referencing have been directly linked to mental health difficulties. Tackman et al. [14], for example, counted references to the self, to we-groups and to other-groups, and found that self-referential language or “I-talk” was a marker for distress, with significant prevalence in individuals with depression. This was taken to reflect the self-rumination and low self-esteem (as I-talk carries minimal social authority) characterising depression (15). We considered references to self to be important to investigate, although in children a self-focus is more typical. But, deictic references are not limited to the person domain. Expressions in domains such as time and space also utilise the same language-context dependencies.

Finally, organisation is considered a fundamental means of structuring our experiences to facilitate reflection, learning, self-knowledge and to make sense of our lives (16–19). A case in point may be the fragmentation of narratives concerning traumatic and difficult to relive events common to those with PTSD (20). In children, narrative organisation has been used to explore inner worlds, regulatory processes but also attachment to others (21–25). Yet, it is unclear what the relationship of organisation is to emotional development in children after adjustment for structural language.

### 1.2. What tools?

To date most of the psychological research on pragmatic language difficulties in children has used the CCC. However, pragmatic skill is very difficult to measure well with standardised tools since these cannot capture social-communication problems that may arise in everyday situations where the rules of engagement are both less explicit and highly dynamic. Some research has used narrative tasks to measure this skill, striking a better balance of naturalism and precision. However, such tasks are typically conversational. This poses a serious limitation because the presence of a conversational partner may provide inadvertent scaffolding (even without them talking) given that the ability to narrativise develops through social interaction or, conversely, may prevent socially anxious individuals from demonstrating their full abilities. Moreover, when studying inherently context-dependent capabilities, investigations across contexts, including that of language (e.g., oral or written), are essential. Therefore, although written tasks may introduce other concerns (such as self-editing), there is arguably much to be learned from a pragmatic analysis of children's written texts.

### 1.3. This study

This study was designed to fill these gaps. Using a large general-population sample in Britain, it performed an analysis of 11-year-old children's written texts for relevance, reference and organisation, and examined how these may be linked to concurrent internalising symptomology as reported by both parents and teachers. The texts were the essays the children were asked to write at age 11 years on how they imagined their lives at age 25 years. It was expected that there would be a link between intact pragmatic language and low levels of internalising symptoms, because, as discussed earlier, (a) good pragmatic skills may facilitate socio-emotional protection by enabling one to express oneself to others and understand them in return, (b) internalising symptoms may result in social withdrawal and therefore poor pragmatic language skills, and (c) poor pragmatic language and poor mental health may share causes such as impaired social cognition, weak executive function but also impoverished social contexts.

## 2. Materials and methods

### 2.1. Sample

The data for this study came from the National Child Development Study (NCDS), a publicly available dataset^1^ (26), which has documented the lives of over 17,000 people, all born in Britain in a single week of March 1958 (27). The cohort was followed at three times in childhood (at ages 7, 11 and 16 years). At age 11, the cohort children were asked to “imagine you are now 25-years-old. Write about the life you are leading, your interests, your home life and your work at the age of 25”^2^. At that age, internalising symptoms were measured by both parent and teacher reports (see Measures). For this study, we randomly selected 100 essays of 11-year-olds whose first language was English from both the highest and lowest 10% of scorers on the parent-reported internalising scale (the most well-validated scale of the two). This decision was made to ensure variability within the sample. The resultant 200 essays were used to blindly evaluate children's three pragmatic language abilities, discussed earlier and described in detail below: relevance, organisation and reference.

### 2.2. Measures

#### 2.2.1. Internalising symptoms

Parent-reported internalising symptoms were measured with Rutter's Neurotic Scale (28). This includes five items scored on 3-point scales detailing whether the child is reluctant to go to school, is miserable or tearful, is often worried, is upset by new situations, and prefers to be alone. Rutter reported test-retest product-moment correlations and inter-rater reliabilities of .89 and .72, respectively. Teacher-reported internalising symptoms were measured with the Bristol Social Adjustment Guide (BSAG), a booklet of 250 descriptors of “maladjusted behaviours”. Each descriptor belonged to one of 12 “syndromes”. Factor analyses of these syndrome scores have repeatedly revealed two-factor solutions corresponding to internalising and externalising scales (29). The internalising scale comprised “withdrawal”, “unforthcomingness”, “depression”, “writing-off adults” and “miscellaneous” symptoms (Cronbach's alpha .73 across the NCDS sample).

#### 2.2.2. Relevance

Relevance was intended to capture the child's comprehension of the question and the appropriateness of their response. The essay title asked for details of their imagined future home-life, work-life and interests. Within each domain we recorded the presence of information of four types: mere facts, elaborative descriptions, own judgments and others' judgments. The inclusion of these categories ensured we could capture the variety of relevant information provided. A maximum of one point was given per type of information when summed per domain, to minimise the advantage of longer essays.

#### 2.2.3. Organisation

Organisation, critical to comprehensible expression, was measured in several ways. Structure, coherence and within-sentence structure variability were judged using a 4-point scale. Structure reflected the sensible separation of sections, or sets of related ideas, e.g., by topic or chronologically, and the consistency of its application. Coherence reflected the linking of ideas or how well the essay flowed. Within-sentence structure variability assessed for varied type, contents and ordering of sentence clauses (e.g., “I will be X. I will have Y” would score poorly). Alongside conjunction word-counts, these were intended to capture the ability to maintain a reader's interest and organise within-sentence.

#### 2.2.4. Reference

Reference was included to capture one's ability to guide a reader's attention. To measure this, length-adjusted word counts were conducted for deictic references, e.g., in “He liked that film”, *he* and *that* may substitute for many referents but index a particular referent through contextual cues. Deictic references were classified into three domains: person, object and time, and, within each domain, into proximal and distal (i.e., within and outside of the deictic centre, respectively). Within the person domain, the proximal group contained first-person pronouns, and the distal group contained second and third-person pronouns. The object domain comprised references to objects, space and the discourse itself; the proximal group in this domain referred to points nearby as in *this* or *here* in contrast to the distal *that* or *there*. The time domain comprised proximal references such as *now* or *today*, and distal references such as *tomorrow*, *next week* or *last year*. Lastly, context-dependent references were also categorised into pure and impure to elucidate how one refers, not just to what. Pure indexicals require no further contextual information to be understood other than their being articulated, e.g., “I” is the speaker/writer and “now” is the present (30). These are invariantly egocentric, or embodied, in that they refer from the default *I, here, now* anchor. This overlaps with but is distinct from content of reference, and thus mental simulation and referent tracking. For instance, tomorrow, whilst referring to a distal point in the future, is defined relative to now and so requires no further information, nor much mental simulation, only knowledge of this interpretive norm, to refer. Conversely, and adapting the term of Kaplan's (30), impure references require further information (and so greater referent-tracking and mental simulation) to instantiate a new non-default, allocentric or disembodied anchor point from which to refer, e.g., “It was nicer in the shade, let's go back (there)”. Other words such as *here* or *later* can be pure or impure, depending on use. (The coding sheet used to derive the study's pragmatic language measures is included in the Supplementary Material).

#### 2.2.5. Confounders

We adjusted for confounding by controlling for the child's sex and cognitive ability [verbal and non-verbal IQ; (31)] but also socioeconomic circumstances and parental education. Specifically, we controlled for maternal education (whether the mother stayed in school to the minimum required age or not), father's socioeconomic group (for those with fathers who were not absent or in the armed forces), maternal employment (whether the mother had worked in the past three years or not) and overcrowding (>1.5 people per room). We also considered important linguistic controls. We therefore further adjusted for (a) mean length of utterance (MLU), measured in morphemes per utterance in the essay); (b) diversity of vocabulary (the mean number of novel word stems per utterance); and (c) the essay word-count. (The python scripts used to measure MLU and diversity of vocabulary are available in the Supplementary Material). Tables 1A and 1B show the distribution of the language and confounding variables in each of the two “Rutter” score deciles, and Table 2 shows their correlations with both “Rutter” and BSAG scores.

**Table 1A:** Continuous variables: descriptive statistics.

Variable Name	Group—Rutter internalising						*t*-test
	Low (bottom decile)			High (top decile)			*p*-value
	Mean (SD)	Min	Max	Mean (SD)	Min	Max	
Verbal IQ	22.52 (9.31)	6	40	19.57 (8.969)	0	37	0.024
Non-verbal IQ	21.08 (6.835)	7	38	18.97 (6.875)	4	34	0.031
BSAG Internalising	3.97 (4.122)	0	16	5.22 (5.183)	0	27	0.061
Rutter Internalising	5.18 (0.386)	5	6	10.8 (0.91)	10	13	<.001
R1—Home (relevance)	1.73 (0.983)	0	4	1.45 (0.989)	0	4	0.046
R2—Work (relevance)	1.77 (0.897)	0	4	1.5 (0.823)	0	4	0.028
R3—Interests (relevance)	1.36 (0.847)	0	3	1.21 (0.808)	0	4	0.202
O1—Structure (organisation)	1.5 (1.193)	−1	3	1.05 (1.038)	−1	3	0.005
O2—Coherence (organisation)	1.59 (1.016)	−1	3	1.09 (0.996)	−1	3	<.001
O3—Variability (organisation)	1.25 (0.989)	−1	3	1.05 (0.947)	−1	3	0.146
O4—Conjunctions (organisation)	9.64 (6.957)	0	34	8.74 (6.276)	0	37	0.338
O3*O4 (organisation)	−0.301 (2.24)	−12.04	7.354	−0.233 (2.661)	−12.857	7.354	0.85
Person—Proximal (reference)	10.884 (3.776)	0	25.806	11.885 (4.169)	4	33.333	0.077
Person—Distal (reference)	1.552 (2.054)	0	13.793	1.426 (1.905)	0	8.756	0.653
Object—Proximal (reference)	0.694 (1.021)	0	5.556	0.509 (0.719)	0	3.158	0.141
Object—Distal (reference)	1.813 (1.74)	0	8.772	1.405 (1.086)	0	4.245	0.048
Time—Proximal (reference)	0.304 (0.563)	0	2.778	0.406 (0.667)	0	3.571	0.245
Time—Distal (reference)	1.391 (1.487)	0	10	1.257 (1.503)	0	8.333	0.527
Pure (reference)	10.9 (4.344)	0	25.806	11.984 (4.553)	0	33.333	0.087
Impure (reference)	5.491 (3.653)	0	18.261	4.602 (3.113)	0	14.747	0.065
MLU	10.705 (3.377)	4.115	23.5	10.222 (2.885)	3	17.333	0.278
Vocabulary diversity	0.642 (0.315)	0.383	3.2	0.652 (0.384)	0.343	4	0.839
Word count	173.45 (114.806)	5	615	155.18(88.322)	3	443	0.209

**Table 1B:** Categorical variables: descriptive statistics.

Variable		Group—Rutter Internalising		Chi square
Name	Category	Low group (bottom decile)	High group (top decile)	*p-*value
		Frequency (*n* = 100)	Frequency (*n* = 100)	
Paternal socioeconomic group (SEG)	1 (highest SEG)	4	3	0.842
	2	9	8	
	4	1	4	
	5	5	5	
	6	10	9	
	8	5	6	
	9	35	35	
	10	13	18	
	11	8	6	
	12	5	1	
	13	1	1	
	14	1	0	
	15 (lowest SEG)	1	2	
	Missing (father in armed forces, or absent, or N/A)	2	2	
Overcrowding (Occupants/room)	Below 1.5	76	83	0.181
	Above 1.5	20	13	
	Missing	4	4	
Maternal education	Compulsory schooling	67	53	0.033
	Did not finish compulsory schooling	31	46	
	Missing	2	1	
Child sex	Male	62	50	0.087
	Female	38	50	
	Missing	0	0	
Maternal employment	Employed in last 3 years	67	59	0.204
	Not employed in last 3 years	32	41	
	Missing	1	0	

**Table 2:** Correlations of study variables with internalising scale scores.

Variable	BSAG internalising	Rutter internalising
	Pearson's *r* (*p*-value)	Pearson's *r* (*p*-value)
BSAG internalising	—	
Rutter internalising	0.14 (0.048)	—
Child sex	−0.226 (0.001)	0.128 (0.07)
Paternal SEG	0.191 (0.008)	−0.029 (0.689)
Maternal education	−0.02 (0.785)	0.148 (0.038)
Maternal employment	−0.069 (0.333)	0.089 (0.213)
Overcrowding	0.221 (0.002)	−0.099 (0.173)
Verbal IQ	−0.214 (0.002)	−0.144 (0.041)
Non-verbal IQ	−0.188 (0.008)	−0.152 (0.031)
Home (relevance)	−0.289 (<.001)	−0.144 (0.041)
Work (relevance)	−0.091 (0.199)	−0.141 (0.047)
Interests (relevance)	−0.136 (0.055)	−0.059 (0.404)
O1—Structure (organisation)	−0.31(<.001)	−0.177 (0.012)
O2—Coherence (organisation)	−0.332(<.001)	−0.232(<.001)
O3 Variability (organisation)	−0.198 (0.005)	−0.101 (0.154)
O4 Conjunctions (organisation)	−0.145 (0.041)	−0.044 (0.533)
O3*O4 (organisation)	−0.1 (0.171)	0.02 (0.787)
Person—Proximal (reference)	0.041 (0.562)	0.127 (0.073)
Person—Distal (reference)	−0.118 (0.096)	−0.053 (0.459)
Object—Proximal (reference)	0.009 (0.903)	−0.106 (0.137)
Object—Distal (reference)	−0.08 (0.264)	−0.147 (0.038)
Time—Proximal (reference)	−0.042 (0.556)	0.063 (0.379)
Time—Distal (reference)	−0.131 (0.065)	−0.026 (0.715)
Pure (reference)	0.047 (0.512)	0.115 (0.104)
Impure (reference)	−0.194 (0.006)	−0.125 (0.079)
MLU	−0.145 (0.041)	−0.093 (0.191)
Vocabulary diversity	0.165 (0.02)	0.0019 (0.998)
Word count	−0.203 (0.004)	−0.079 (0.264)

### 2.3. Analytic strategy

First we tested the reliability of the language measures, using three raters, blind to children's internalising scores or any demographic characteristic, for 20 essays (10% of the sample). The inter-rater reliability was assessed using the intra-class correlation (ICC). After discussion, mean reliabilities of 88.8% across relevance and 81% across organisation were observed. For word-count measures (reference variables and conjunctions) a reliability of 96.5% was observed, thus no amendments were made after discussion. Overall, mean ICC across measures was 91.5%, indicating good reliability.

Then we examined the relationship between the two internalising symptom scales. Their correlation was modest (*r* = .14, *p* = .048). This difference may result from the two scales' distinct foci and contexts. The “Rutter” scale items are parents' assessments of their children's emotional states and behaviours whereas the teacher-reported BSAG may emphasise children's social functioning given that it considers only behaviours observed in a social setting (school).

Last we fitted multiple linear regression models to examine the prediction of internalising symptom scores from the three pragmatic language measures, after adjustment for confounders. First, the control model (including just confounders) was fitted. Next, the pragmatic language variables were added. Then, significant individual predictors were re-modelled alongside the linguistic controls. The next section presents the regression results for the BSAG. For brevity, only the differences in the results obtained using the “Rutter” scale are reported, at the end of the section.

## 3. Results

Using the BSAG, the regression model including only confounders (the control model) was significant, *F*(7, 183) = 4.329, *p* < .001, *R*^2^ = .142. Results of all regression models and for each internalising symptom scale are presented in Tables 3, 4. Inspection of the variance inflation factor indicated that multicollinearity was not detected in any model.

**Table 3:** Multiple linear regression results for teacher-rated internalising symptoms (BSAG scores).

Step	*F* ratio	*p*	*R* ^2^	adj *R*^2^	Variables	*b*	S.E.	*β*	*t*	Sig
1	*F*(7, 180) = 4.267	<0.001	0.142	0.109	Child sex	−1.931	0.695	−0.204	−2.78	0.006
					Maternal education	0.574	0.936	0.048	0.614	0.54
					Paternal SEG	0.105	0.121	0.068	0.862	0.39
					Maternal employment	−0.448	0.687	−0.046	−0.652	0.515
					Overcrowding	2.357	0.893	0.189	2.638	0.009
					Verbal IQ	−0.019	0.058	−0.037	−0.323	0.747
					Non-verbal IQ	−0.085	0.076	−0.125	−1.126	0.262
2	*F*(10, 177) = 3.727	<.001	0.174	0.127	R1 (home)	−0.759	0.361	−0.16	−2.101	0.037
					R2 (work)	−0.247	0.42	−0.045	−0.587	0.558
					R3 (interests)	−0.347	0.417	−0.061	−0.833	0.406
3	*F*(13, 174) = 2.846	.001	.175	.114	R1 (home)	−0.672	0.413	−0.142	−1.625	0.106
					R2 (work)	−0.172	0.499	−0.032	−0.344	0.731
					R3 (interests)	−0.26	0.486	−0.046	−0.535	0.593
					MLU	−0.084	0.312	−0.056	−0.271	0.787
					Vocabulary diversity	0.178	0.583	0.057	0.306	0.76
					Word count	−0.001	0.007	−0.016	−0.104	0.917
										
2	*F*(8, 166) = 4.332	<.001	0.173	0.133	O1 (structure)	−0.989	0.401	−0.194	−2.469	0.015
3	*F*(11, 163) = 3.309	<.001	.183	.127	O1 (structure)	−0.866	0.43	−0.17	−2.014	0.046
					MLU	−0.175	0.318	−0.117	−0.55	0.583
					Vocabulary diversity	0.363	0.595	0.117	0.611	0.542
					Word count	−0.002	0.006	−0.038	−0.282	0.778
2	*F*(8, 172) = 4.824	<.001	0.183	0.145	O2 (coherence)	−1.182	0.402	−0.219	−2.936	0.004
3	(*F*(11, 169) = 3.564	<.001	.188	.135	O2 (coherence)	−1.082	0.465	−0.201	−2.33	0.021
					MLU	−0.157	0.311	−0.105	−0.504	0.615
					Vocabulary diversity	0.37	0.581	0.119	0.636	0.526
					Word count	0	0.006	−0.005	−0.037	0.971
2	*F*(10, 171) = 3.077	0.001	0.152	0.103	Centred O3 (variability)	−0.434	0.443	−0.078	−0.979	0.329
					Centred O4 (conjunctions)	−0.093	0.133	−0.061	−0.695	0.488
					Centred O3*O4	−0.2	0.171	−0.104	−1.171	0.243
2	*F*(12, 161) = 3.577	<.001	0.203	0.144	O1 (structure)	−0.567	0.493	−0.111	−1.15	0.252
					O2 (coherence)	−1.222	0.574	−0.227	−2.129	0.035
					Centred O3	0.592	0.549	0.106	1.08	0.282
					Centred O4	−0.085	0.133	−0.056	−0.638	0.525
					Centred O3*O4	−0.215	0.17	−0.112	−1.263	0.208
3	*F*(15, 159) = 2.921	<.001	.216	.142	O1 (structure)	−0.594	0.496	−0.117	−1.198	0.233
					O2 (coherence)	−1.159	0.584	−0.215	−1.983	0.049
					Centred O3	0.937	0.612	0.168	1.53	0.128
					Centred O4	−0.109	0.135	−0.071	−0.809	0.42
					Centred O3*O4	−0.237	0.172	−0.123	−1.377	0.171
					MLU	−0.137	0.316	−0.091	−0.432	0.667
					Vocabulary diversity	0.381	0.593	0.122	0.642	0.522
					Word count	−0.594	0.496	−0.117	−1.198	0.233
2	*F*(9, 178) = 3.490	0.001	0.15	0.107	Person—Proximal	0.021	0.089	0.018	0.24	0.811
					Person—Distal	−0.196	0.181	−0.082	−1.083	0.28
2	*F*(9, 178) = 3.358	0.001	0.145	0.102	Object—Proximal	0.09	0.376	0.017	0.238	0.812
					Object—Distal	−0.171	0.231	−0.053	−0.74	0.46
2	*F*(9, 178) = 3.710	<.001	0.158	0.115	Time—Proximal	−0.476	0.548	−0.062	−0.868	0.386
					Time—Distal	−0.36	0.22	−0.114	−1.64	0.103
2	*F*(9, 178) = 3.725	<.001	0.159	0.116	Proximal (total)	0.004	0.267	0.001	0.013	0.989
					Distal (total)	−0.575	0.328	−0.13	−1.755	0.081
2	*F*(9, 178) = 3.921	<.001	0.165	0.123	Pure (total)	−0.01	0.078	−0.01	−0.128	0.898
					Impure (total)	−0.221	0.105	−0.16	−2.112	0.036
3	*F*(12, 175) = 3.088	<.001	.175	.118	Pure (total)	−0.022	0.079	−0.021	−0.279	0.78
					Impure (total)	−0.178	0.11	−0.129	−1.614	0.108
					MLU	−0.117	0.308	−0.078	−0.38	0.705
					Vocabulary diversity	0.209	0.572	0.067	0.365	0.716
					Word count	−0.003	0.006	−0.067	−0.517	0.606

**Table 4:** Multiple linear regression results for parent-rated internalising symptoms (“Rutter” scores).

Step	*F* ratio	*p*	*R* ^2^	adj *R*^2^	Variables	*b*	S.E.	*β*	*t*	Sig
1	*F*(7, 180) = 3.177	0.003	0.11	0.075	Child sex	0.841	0.435	0.144	1.931	0.055
					Maternal education	1.384	0.587	0.189	2.359	0.019
					Paternal SEG	−0.128	0.076	−0.135	−1.677	0.095
					Maternal employment	0.833	0.431	0.14	1.935	0.055
					Overcrowding	−1.161	0.56	−0.151	−2.073	0.04
					Verbal IQ	−0.033	0.037	−0.107	−0.916	0.361
					Non-verbal IQ	−0.031	0.048	−0.074	−0.652	0.515
2	*F*(10, 177) = 3.020	0.001	0.146	0.098	R1 (home)	−0.54	0.226	−0.185	−2.388	0.018
					R2 (work)	−0.248	0.263	−0.074	−0.945	0.346
					R3 (interests)	0.06	0.261	0.017	0.232	0.817
3	*F*(13, 174) = 2.32	.007	.148	.084	R1 (home)	−0.597	0.259	−0.204	−2.309	0.022
					R2 (work)	−0.301	0.313	−0.09	−0.962	0.337
					R3 (interests)	0.004	0.304	0.001	0.012	0.991
					MLU	0.018	0.195	0.019	0.092	0.927
					Vocabulary diversity	−0.075	0.364	−0.039	−0.206	0.837
					Word count	0.001	0.004	0.041	0.267	0.79
2	*F*(8, 165) = 4.138	<.001	0.166	0.126	O1 (structure)	−0.829	0.248	−0.265	−3.348	0.001
3	*F*(11, 163) = 2.964	.001	.167	.110	O1 (structure)	−0.821	0.267	−0.262	−3.071	0.002
					MLU	−0.053	0.198	−0.057	−0.266	0.79
					Vocabulary diversity	0.08	0.37	0.041	0.215	0.83
					Word count	0.001	0.004	0.02	0.144	0.886
2	*F*(8, 172) = 4.215	<.001	0.164	0.125	O2 (coherence)	−0.835	0.251	−0.252	−3.332	0.001
3	*F*(11, 169) = 3.031	<.001	.165	.110	O2 (coherence)	−0.887	0.29	−0.267	−3.06	0.003
					MLU	−0.032	0.194	−0.035	−0.167	0.868
					Vocabulary diversity	0.065	0.363	0.034	0.178	0.859
					Word count	0.001	0.004	0.05	0.367	0.714
2	*F*(10, 171) = 2.36	0.012	0.121	0.07	Centred O3 (variability)	−0.344	0.277	−0.1	−1.241	0.216
					Centred O4 (conjunctions)	0.025	0.084	0.027	0.299	0.765
					Centred O3*O4	0.068	0.107	0.057	0.636	0.526
2	*F*(12, 162) = 3.070	<.001	0.185	0.125	O1 (structure)	−0.563	0.307	−0.18	−1.834	0.069
					O2 (coherence)	−0.669	0.357	−0.201	−1.872	0.063
					Centred O3	0.33	0.341	0.096	0.967	0.335
					Centred O4	0.028	0.083	0.029	0.333	0.739
					Centred O3*O4	0.056	0.106	0.047	0.529	0.597
3	*F*(15, 159) = 2.417	.003	.186	.109	O1 (structure)	−0.569	0.311	−0.182	−1.829	0.069
					O2 (coherence)	−0.675	0.367	−0.203	−1.842	0.067
					Centred O3	0.307	0.384	0.089	0.799	0.425
					Centred O4	0.027	0.085	0.029	0.321	0.749
					Centred O3*O4	0.056	0.108	0.047	0.518	0.605
					MLU	−0.049	0.198	−0.053	−0.245	0.807
					Vocabulary diversity	0.095	0.372	0.05	0.256	0.798
					Word count	0.001	0.004	0.038	0.258	0.796
2	*F*(9, 178) = 2.739	0.005	0.122	0.077	Person—Proximal	0.049	0.056	0.068	0.882	0.379
					Person—Distal	−0.099	0.113	−0.068	−0.875	0.383
2	*F*(9, 178) = 2.911	0.004	0.128	0.084	Object—Proximal	−0.3	0.234	−0.091	−1.281	0.202
					Object—Distal	−0.199	0.143	−0.1	−1.389	0.166
2	*F*(9, 178) = 2.708	0.006	0.12	0.076	Time—Proximal	0.49	0.344	0.104	1.422	0.157
					Time—Distal	−0.033	0.138	−0.017	−0.238	0.812
2	*F*(9, 178) = 2.82	0.004	0.125	0.081	Proximal (total)	0.12	0.165	0.055	0.726	0.469
					Distal (total)	−0.26	0.206	−0.096	−1.266	0.207
2	*F*(9, 178) = 2.837	.004	0.118	0.074	Pure (total)	0.034	0.049	0.052	0.688	0.493
					Impure (total)	−0.084	0.066	−0.099	−1.275	0.204
3	*F*(12, 175) = 2.138	.017	.128	.068	Pure (total)	0.028	0.05	0.043	0.561	0.576
					Impure (total)	−0.071	0.07	−0.084	−1.025	0.307
					MLU	−0.006	0.195	−0.007	−0.033	0.974
					Vocabulary diversity	−0.054	0.362	−0.028	−0.149	0.881
					Word count	−0.001	0.004	−0.049	−0.364	0.716.

For relevance, the three domains (home, work and interests) were entered together as distinct predictors, since information in one domain may constrain the space for information in another. The model was significant [*F*(10, 177) = 3.727, *p* < .001], contributing Δ*R*^2^ = .032 above controls. Only relevant information to home life was negatively associated with internalising scores, *β* = −.16, *p* = .037, suggesting that children with more internalising symptoms provide little or less varied information pertaining to home life. However, after the introduction of linguistic controls, the provision of relevant home-life information was no longer significant, with *β* = −.14, *p* = .106 (although it was still significant in the model predicting parent-rated symptoms). This indicates an overlap between communicating relevant information and structural language skills.

For organisation, the four measures were entered first individually and then amongst other measures of organisation. First, structure contributed Δ*R*^2^ of .03 towards the model's explained variance, and uniquely predicted fewer internalising symptoms both without (*β* = −.19, *p* = .015) and with linguistic controls (*β* = −.17, *p* = .046). Next, coherence was modelled. This added Δ*R*^2^ of .041 to the model's explained variance beyond the control-model, predicting fewer internalising symptoms both without (*β* = −.22, *p* = .004) and alongside linguistic controls (*β* = −.20, *p* = .021). In contrast, neither variability of sentence structure nor number of conjunctions within-sentence uniquely predicted internalising scores in any model. Their interaction—capturing, for example, essays with more conjunctions in varied structures—was thought to provide a more balanced measure of within-sentence organisational skill. However, no significant unique predictors were found in a model of these when centred and entered with their interaction, contributing Δ*R*^2^ = .01 variance above controls, *F*(10, 171) = 3.077, *p* = .001. A full model of organisation was then fitted. This contributed Δ*R*^2^ = .09 with *F*(12, 161) = 3.577, *p* < .001. In this full model, coherence again predicted fewer internalising symptoms, *β* = −.23, *p* = .035, but no other organisation variable did so, and, after the addition of the linguistic control variables, coherence remained the only significant unique predictor (*β* = −.22 *p* = .049). This suggests a degree of shared variance between measures of organisation, and highlights that coherence is the aspect of organisation that is most pertinent to internalising symptoms.

For reference, we compared first the predictive validity of proximal and distal deictic references with separate models per domain (of person, object and time). In these domain-specific models, there were no significant unique predictors of internalising scores. Even when summed across domains, the total proximal and distal references did not reach significance. Next, we categorised deictic references into pure and impure, rather than by content. This model explained Δ*R*^2^ = .023 above controls, with *F*(9, 178) = 3.921 and showed that impure references were negatively associated with internalising symptoms, with *β* = −.16, *p* = .036. However, in the presence of linguistic controls impure references no longer predicted internalising symptoms, with *β* = −.129, *p* = .108. This suggests that referring to objects, people and time using non-default anchor points was associated with fewer internalising symptoms but not beyond other linguistic abilities.

Results using the parent-reported internalising symptoms were broadly similar. There were two exceptions in the models of the “Rutter” scores. First, the significant effect of narrative coherence was not evident in the combined organisation model. Second, there was a unique effect of variety of relevant information concerning home life. The greater focus of the BSAG on social behaviour may be relevant to these differences, as discussed.

## 4. Discussion

This study analysed the essays written by a sample of the 1958 British birth cohort when they were aged 11 years on their imagined home and work lives as adults. Using a novel approach to measuring three pragmatic language features of the children's written texts (relevance, organisation and deictic references) it demonstrated important associations between them and children's internalising symptoms as rated by their parents and teachers.

The inverse association we found between relevance and parent-reported internalising symptoms suggests the importance of communicative capacities in children's social and emotional lives. The use of relevant language pertaining especially to home life was consistently associated with lower symptom scores in our sample. These between-domain differences may reflect the centrality of the home and family to children's lives. Previous research for example has documented the poor-quality representation of caregivers in the narratives of children facing emotional difficulties (25, 32, 33). Moreover, a study also using the written essays at age 11 years of the 1958 British birth cohort found that descriptions of family were correlated with improved mental health (34). Thus, among children, a lack of attention to their future home-life in their essays may reflect discontentment with their present one. Moreover, since a child's home is inherently social (as no child lives alone), discussions of home life may naturally also capture the degree of the child's attention to and understanding of others—which discussions of future work and future leisure activities may do to a lesser extent.

With respect to organisation, we showed that, when considered separately, structure and coherence were negatively related to internalising symptom scores beyond confounders. But, coherence did so even in the presence of other organisational measures. We expected that structure, indexing reasonable ordering, would be inversely associated with internalising symptoms even when considered alongside coherence. We note however that in our study a high-level structure was primed by the essay question listing the life domains (home life, work and interests) the children were to cover. It would be interesting to know what links would remain without such a prime. The robust effect of coherence on the other hand was in line with our expectations. If Wittgenstein's (35) and Frege’s (36) claim that sentences are the most basic unit of meaning were accepted—e.g., even “stop!” is parasitic on the fuller “stop what you are doing”—it would follow that coherence, i.e., effective organisation between sentences, is most fundamental to maintaining successful social interactions.

Finally, with respect to reference, we found that impure references predicted fewer internalising symptoms before adding linguistic controls. This highlighted that increased simulational demands and the tracking of contextual information necessary to articulate referential anchor shifts in communication were inversely related to internalising symptoms. This association was robust to adjustment for cognitive ability but was explained away by structural language. In our study, structural language (i.e., MLU, diversity of vocabulary and word count) difficulties likely captured expressive disengagement through withdrawal, lethargy and loss of motivation typically associated with depressive symptoms. In turn, this suggests important links between poor referential, grammatical and lexical abilities and depressive and anxiety symptoms in the general child population, which ought to be investigated further.

Overall, it appeared that communicative skills—such as coherence, varied and relevant responses and broad referential range—were inversely related to internalising symptoms in children even after controls for socio-demographic background and cognitive ability. Most of these associations however were confounded by structural language, although two effects did remain. First, children providing varied and relevant information about their future home-life had lower parent-reported internalising symptoms. This suggests that children's narratives of family life could provide a useful measure of emotional state. Second, coherence was inversely related to teacher reports of internalising symptoms. Given that longitudinal studies in childhood have observed influences from language difficulties to internalising symptoms, not the reverse (6), this suggests that coherent communication may provide a protective buffer to emotional difficulties. Although our cross-sectional study did not allow us to test for mechanisms underlying such a relationship we argue that coherence—i.e., the child's ability to convey a logical sequence of events, provide the right amount of key information, and produce meaningful content that is free from unusual or bizarre expressions—is likely instrumental for high-quality social interactions (7), in turn lowering the risk of depressive and anxiety symptoms.

## 5. Conclusion

This study analysed essays written by a sample of the 1958 British birth cohort when they were aged 11 years on their imagined home and work lives as adults. It used a novel approach to measure three pragmatic language features (relevance, organisation and deictic references) and associated them with children's concurrent internalising symptoms, as reported by their parents and teachers. For relevance, the variety of pertinent information concerning home life uniquely predicted fewer internalising symptoms as reported by the children's parents. Organisational skill was measured at three levels, of which coherence between sentences was the most consistent predictor of low internalising symptoms; more so than discourse and within-sentence organisation. Finally, children making more use of reference shifts (from the speaker, time or place of utterance to some other object, time or place) had lower internalising symptom scores but this association was explained away by structural language, suggesting that linguistic competence was a confounder. Our findings are noteworthy but preliminary. It will be important to test and refine our coding scheme with contemporary child samples and at various stages of development.

## Data Availability

The raw data supporting the conclusions of this article will be made available by the authors, without undue reservation.

## Footnotes

^1^
https://beta.ukdataservice.ac.uk/datacatalogue/series/series?id=2000032.

^2^
For examples of the essays please see https://cls.ucl.ac.uk/cls-studies/1958-national-child-development-study/ncds-age-11-sweep/age-11-essays/, and for the data guide please see http://doc.ukdataservice.ac.uk/doc/5790/mrdoc/pdf/ncds_age_11_essays_user_guide.pdf.
