# Supplementary material for: Poor written pragmatic skills are associated with internalising symptoms in childhood: evidence from a UK birth cohort study
Source: Front Child Adolesc Psychiatry. 2023 Jun 14;2:1075836. doi: 10.3389/frcha.2023.1075836 (PMC11731994; doi:10.3389/frcha.2023.1075836)
Supplement: Supplementary file 1 [file Datasheet1.docx]

Supplementary Material

# Data access

The full NCDS dataset can be accessed from the UK data service website, via <https://beta.ukdataservice.ac.uk/datacatalogue/series/series?id=2000032>.

A detailed guide to the child essays at age 11 years in NCDS is available, via <http://doc.ukdataservice.ac.uk/doc/5790/mrdoc/pdf/ncds_age_11_essays_user_guide.pdf>.

A selection of the child essays in NCDS is available to view, via <https://cls.ucl.ac.uk/cls-studies/1958-national-child-development-study/ncds-age-11-sweep/age-11-essays/>

# Coding sheet

- *Relevance*
- *Note: a maximum of one can be scored for each criterion. However, if one category conflates with another, i.e. their interest seemingly is their job, then possible marks for each domain may be added together. This should be noted under comments.*
  - **R1** - Is home life mentioned?
    - 0 - no
    - 1 – mere facts, basic description e.g. *where they live and who with*
    - 1 – elaborative description beyond facts, at least 2 sentences* articulating what home life is like e.g. *I live in a great big house overlooking the country, not so far from where my grandparents live.*
    - 1 – their own judgment or evaluations, e*.g. I am happily married*
    - 1 – judgement of others, e.g. *my kids love playing in the garden*
  - **R2** - Is work life mentioned?
    - 0 - no
    - 1 – mere facts e.g., *what they do, where work is*
    - 1 – elaborative description, at least 2 sentences* articulating*. what happens at work, what’s it like?*
    - 1 – their own judgment or evaluations
    - 1 – judgement of others
  - **R3** - Are interests mentioned?
    - 0 - no
    - 1 – mere facts, e.g. I like cycling and football
    - 1 – elaborative description, at least 2 sentences* articulating *what about the interests they like, when they do them, who with, etc*
    - 1 – their own judgment or evaluations
    - 1 – judgement of others
- *Organisation*
  - **O1** – Structure *(Are there clear sections relating to topic, theme, or time)*
    - 3 – consistently (all information presented in distinct sections, or logical sequence)
    - 2– mostly (related information mostly in sections, or in sequence, requires at least 3 sections)
    - 1 – somewhat (information ordering is inconsistent)
    - 0– no (no ordering of information)
    - -1 – NA (under 3 sentences or just 1 section)
  - **O2** – Coherence (Do sentence topics follow from one another, are events and information linked).
    - 3 – consistently (information flows consistently, no abrupt topic shifts)
    - 2 – frequently (information mostly related and reading is mostly smooth)
    - 1 – occasionally (some attempts to link information, abrupt topic changes are common)
    - 0 – Seldom – (almost no relation between sentences)
    - -1- NA (under 3 sentences)
  - **O3** – Are sentence structures varied? *Consider* ***variety*** *in sentence length, subject changes, presence of clauses and adverbs.*
    - 3 – consistently (variety in sentence structure almost constant)
    - 2 – frequently (sentence structure varies most of the time)
    - 1 – occasionally (sentence structure noticeably repetitive)
    - 0 – Seldom – (almost always repeats sentence structures)
    - -1 – NA (under 3 sentences)
  - **O4** - Number of conjunction words (within sentences, between clauses)

** Or independent clauses*

- *Reference*
- Here we are looking at the explicit context-dependent (deictic) labelling of referents, rather than any instances of reference. This means only words that refer from a particular point, such that if the context of utterance were to change so would the meaning. Typically, a first introduction to a referent will not be counted, then references back or forward will be counted. *E.g. I went to* ***the park****. I played on the swings* ***there****.*  The use of ‘*there*’ would be counted as an object reference. ‘The park’ is introduced correctly but will not be counted in this analysis. This is primarily to help discriminate referential ability and to avoid losing differences in the data to noise.
  - Person *–pronouns, determiners (I, you, he, she, we, my, our)*
    - Number of proximal references (I, we, us, in deictic centre)
    - Number of distal references (to outside of deictic centre, not I, we, us)
  - Object *– object, space, discourse, social (it, this, that, here, there, the former)*
    - Number of proximal references (here, this, in deictic centre)
    - Number of distal references (to outside of deictic centre)
  - Time – *tomorrow, next year, now*
    - Number of proximal references (now, in deictic centre)
    - Number of distal references (to outside of deictic centre)
  - Impure references – *He, she, it, this, that,* *
    - An indexical referent that would not make sense without accompanying information or demonstration, non-default (contextually established) deictic centre
  - Pure references – *I, today, tomorrow, actual **
    - A reference that makes sense without further information, demonstration, i.e,. its context-referent relation is consistent across situations (I= the speaker), utilises default deictic centre

** Here, now and others can be either pure or impure depending on use (deictic anchor shifting from default or assumed centre)*

# Python Code for MLU and Vocabulary Diversity Variables

#------------------------------------------------------------------------------------------------------------- #

#------------------------------------------------------------------------------------------------------------- #

#--- SETUP ---

# Import packages and libraries

import spacy

nlp = spacy.load('en')

from spacy import displacy

import xlsxwriter

import os

# set directory

directory = '/Users/folderWithNCDSEssays/'

os.chdir(directory)

# create global workshet

workbook = xlsxwriter.Workbook('compcodedmlusentences.xlsx')

worksheet = workbook.add_worksheet()

SetupWorksheet()

# global variables -reset for new batch of essays

StudentCounter = 1

#---RUN LOOP FOR SAMPLE FILES---

for filename in os.listdir(directory):

print(StudentCounter)

if filename.endswith(".rtf"):

with open(filename, encoding = "ISO-8859-1") as f:

doc = nlp(f.read())

ProcessData(doc)

StudentCounter += 1

continue

else:

continue

workbook.close()

#---FUNCTIONS---

#load before running loop

def SetupWorksheet():

bold = workbook.add_format({'bold': True})

row = 0

worksheet.write(row, 0, 'Student ID', bold)

worksheet.write(row, 1, 'Number of Sentences', bold)

worksheet.write(row, 2,'Number of Tags', bold)

worksheet.write(row, 3,'Number of Unique Tags', bold)

worksheet.write(row, 4, 'Number of Lemmas', bold)

worksheet.write(row, 5, 'Number of Unique Lemmas', bold)

def ProcessData(doc):

reset()

StudentID = GetStudentID(doc)

# visualise(doc)

numSentences = GetNumSentences(doc)

numTags = GetNumTags(doc)

numUniqueTags = GetNumUniqueTags(doc)

numLemmas = GetNumLemmas(doc)

numUniqueLemmas = GetNumUniqueLemmas(doc)

content = [StudentID, numSentences, numTags, numUniqueTags, numLemmas, numUniqueLemmas]

WriteExcelData(content)

#pre-process

def reset():

# for new file reset variables

sentences = []

lemmas = []

uniquelemmas = []

morphemes = []

# with open('doc') as f:

# doc = nlp(f.read())

def visualise(doc):

options = {"compact": True, "bg": "#09a3d5",

"color": "white", "font": "Source Sans Pro"}

sentence_spans = list(doc.sents)

displacy.serve(sentence_spans, style="dep")

def GetStudentID (doc):

StudentID = doc[2].text

return StudentID

def TrimDoc(doc):

doc2 = doc[6:-4]

return doc2

def GetNumSentences(doc):

sentences = list(doc.sents) # list of all sentences

return len(sentences)

def GetNumTags(doc):

#use fine grained part of speech tags

tags = doc.count_by(spacy.attrs.TAG)

totalNumTags = 0

for x, y in sorted(tags.items()):

totalNumTags += y

return totalNumTags

def GetNumUniqueTags(doc):

# to be divided by number of sentences to derive MLU estimates

morphemes = doc.count_by(spacy.attrs.TAG)

return len(morphemes)

def GetNumLemmas(doc):

lemmas = list()

for token in doc:

lemmas.append(token.lemma) #make a list of all lemmas

return len(lemmas)

def GetNumUniqueLemmas(doc):

# to be divided by number of sentences to derive vocab estimates'

lemmas = list()

uniquelemmas = list()

for token in doc:

lemmas.append(token.lemma)

if token.lemma not in uniquelemmas:

uniquelemmas.append(token.lemma) #make a list of all unique lemmas

return len(uniquelemmas)

def WriteExcelData(content):

row = StudentCounter

column = 0

for item in content:

worksheet.write(row, column, item)

column += 1

#------------------------------------------------------------------------------------------------------------- #

#------------------------------------------------------------------------------------------------------------- #
